# Diels–Alder-Crosslinked Polymers Derived from Jatropha Oil

**DOI:** 10.3390/polym10101177

**Published:** 2018-10-22

**Authors:** Muhammad Iqbal, Remco Arjen Knigge, Hero Jan Heeres, Antonius A. Broekhuis, Francesco Picchioni

**Affiliations:** 1 Department of Chemistry, Institut Teknologi Bandung, Jalan Ganesha No. 10, 40132 Bandung, Indonesia; iqbal@chem.itb.ac.id; 2 ENgineering and TEchnology Institute Groningen (ENTEG), Chemical Product Engineering, University of Groningen, Nijenborgh 4, 9747 AG Groningen, The Netherlands; remco.knigge@gmail.com (R.A.K.); H.J.Heeres@rug.nl (H.J.H.); a.a.broekhuis@rug.nl (A.A.B.)

**Keywords:** fatty acid methyl ester, jatropha oil, epoxidation, polyketone, thermoreversible

## Abstract

Methyl oleate, methyl linoleate, and jatropha oil were fully epoxidized using in situ-generated performic acid. The epoxidized compounds were further reacted with furfurylamine in a solvent-free reaction to obtain furan-functionalized fatty esters which, then, functioned as oligomers for a network preparation. Thermoreversible crosslinking was obtained through a (retro) Diels–Alder reaction with bismaleimide, resulting in the formation of a brittle network for furan-functionalized methyl linoleate and jatropha oil. The furan-functionalized fatty esters were mixed with alternating (1,4)-polyketone reacted with furfurylamine (PK-Furan) for testing the mechanical and self-healing properties with DMTA and DSC, respectively. Full self-healing properties were found, and faster thermoreversibility kinetics were observed, compared to PK-Furan.

## 1. Introduction

Thermosetting polymers are widely used in industry, due to their tunable mechanical properties (modulus, strength, and durability) as well as their chemical and thermal resistance. This is due to the relatively high crosslinking density displayed by these materials. However, the same factor, i.e., high crosslinking density, hampers the possibility for these materials to be reshaped or recycled. This is related to the factual impossibility of selectively cleaving the crosslinking bonds without affecting the main chain ones. As a consequence, end-of-life thermosets can be recycled only according to a “cradle-to-grave” approach (e.g., by incineration), thus adding pollution to the environment. Another drawback of thermosets is the dependence on oil, the main sources for raw materials of this class of chemical products. In the long term, these factors render thermoset polymers unfit, in a general context, as sustainable (chemical) products [1,2].

Recycling of thermosets has been an important field in sustainability research, since it may decrease the need for raw materials and significantly lower the ecological impact. The main difficulties are the development of efficient processes for the recovery of the starting materials and the worse mechanical properties upon recycling [3]. The use of reversible crosslinking bonds might represent a solution to this problem. By disassembling the polymer network, the material could be recovered and used in other products of similar value and applications (i.e., according to a “cradle-to-cradle” approach).

Different approaches of obtaining reworkable thermosets properties have already been investigated. One of most popular approaches is the use of thermally reversible crosslinked polymers. This concept involves a crosslinking reaction at relatively low temperatures (40–50 °C), while the reverse reaction (i.e., the disassembling of the network) occurs at higher temperatures (110–140 °C) [4]. Crosslinked polymers can be prepared using a Diels–Alder (DA) cycloaddition of furan and maleimide, giving a reversed reaction through a retro Diels–Alder (rDA). For instance, self-healing thermosets, based on polymerization through Diels–Alder crosslinking with a tetra-methoxyfuran monomer and 1,8-bis(maleimide)-1-ethylpropane, have already been reported [5]. Likewise, thermally reversible reactions, based on furan-functionalized polyketone and bismaleimide through DA and rDA, have already been reported, with excellent efficiency for thermosets self-healing [6,7].

While the approaches described above might provide a sustainable solution for the end-of-life of thermosetting polymers, the use of renewable resources for (total or partial) substitution of thermosets raw material might also constitute another added value. Indeed, renewable resources are more environmentally friendly and, also, interesting from an economic point of view (i.e., low cost and abundance). Vegetable oils are considered as one of the potential sources for biopolymers, due to their unsaturated fatty esters structures which contains double bonds (for possible functional group transformation) [8]. Jatropha oil (JO) is considered as a promising source for polymer, not only because of its high unsaturated fatty acid content (3.2–3.8 double bonds per triglyceride molecule) but, also, due to its toxic nature of common variety (no direct competition with the food sector) [9]. Even though JO is mainly investigated for biofuel production, as a great potential replacement to diesel, the oil is potentially developed for other types of applications (e.g., lubricant, pharmaceutical, polymer, etc.) [10,11,12,13,14].

Regarding the potentiality of JO in polymer applications, several synthetic routes have been reported in literature, in order to obtain a vegetable oil-based polymer [8]. Some popular routes involve the modification of the reactive functional groups on the fatty chains (i.e., double bonds and ester group), in order to yield a molecule with desired functionalities (e.g., hydroxyl, oxirane ring, etc.). A high-yield epoxidation method of vegetable oils has been reported, using in situ-generated performic acid [15]. In addition, kinetic studies on epoxidation of JO, using peroxyacetic and peroxyformic acid, have also been reported [16]. In terms of improving the versatility of triglycerides, the epoxidation process is considered to be an important reaction route, since the oxirane ring is much more reactive, and opens the possibility for reaction with various nucleophiles [8]. As an example is the aniline functionalization of epoxidized methyl oleate, catalyzed by an ionic liquid [17]. Similarly, addition of acetic anhydride to methyl oleate has also been reported [18]. A promising salt-catalyzed aminolysis reaction has also been widely studied, for instance, the use of LiBr [19] and, also, ZnCl_2_ [20], for epoxide ring opening using several different aliphatic and aromatic amines. An interesting result was reported regarding the reactions of epoxidized triglyceride with *n*-hexylamine and 1,6-hexamethylenediamine. Instead of only aminolysis, the reaction also shows amidation as a primary reaction, which leads to crosslinking products [21]. However, the crosslinked product was not explored any further.

Based on previously reported results, the research strategy of this work is divided into several steps. The first step involves the chemical modification of the double bonds on unsaturated fatty ester chains into epoxy groups. The second one is the reaction of furfurylamine with epoxidized fatty esters, yielding a furan-modified monomer. Thirdly, the polymerization of the furan-functionalized monomers with bismaleimide via DA reaction, followed by characterization as a potentially thermoreversible polymer. Last but not least, the use of the resulted polymer as a partial substitute in the well-established thermoreversible system of crosslinked furan-functionalized alternating polyketone. To the best of our knowledge, the second, third, and fourth steps of the research have not been previously reported in the literature.

## 2. Materials and Methods

### 2.1. Materials

Methyl oleate (methyl *cis*-9-octadecenoate ≥99%, Sigma-Aldrich, Munich Germany), methyl linoleate (methyl *cis*,*cis*-9,12-octadecadienoate ≥99%, Sigma-Aldrich), and jatropha oil (3.3 double bonds per triglyceride units, locally hot-pressed from *Jatropha curcas* L. seeds, Cape Verde) are used as sources of fatty acid moieties. For the epoxidation reaction, formic acid (ACS Reag PH Eur ≥99%, Merck Chemicals, Darmstadt, Germany), hydrogen peroxide (stabilized, Ph Eur, BP, USP 30%, Merck Chemicals), and benzene (ReagentPlus^®^, thiophene free, ≥99%, Sigma-Aldrich) were used as received. Trimethylsulfonium hydroxide (derivatization reaction for GC, 0.25 M in methanol, Fluka) and *tert*-butyl ether (analytical standard, Sigma-Aldrich) are used for fatty esters analysis using GC-MS. LiBr (anhydrous, ≥99%, Sigma-Aldrich) was used to assist the furan functionalization reaction of epoxidized compound using furfurylamine (2-aminomethylfuran ≥99%, Sigma-Aldrich) and bismaleimide for the crosslinking reaction (1,1′-(methylenedi-4,1-phenylene)bismaleimide ≥95%, Sigma-Aldrich), and were used without further purification. In addition, THF (anhydrous, BHT inhibitor, ≥99.9%, Sigma-Aldrich) and chloroform (anhydrous, PCR reagent, amylene stabilizer, ≥99% Lab-Scan, Munich, Germany) were used as solvent. Chloroform-d (99.96 atom% D, Sigma-Aldrich) and DMSO-d_6_ (99.96 atom% D, Sigma-Aldrich) were used for NMR analysis.

### 2.2. Epoxidation Reaction

The epoxidation reaction was adapted from previously reported works [15]. Two-step epoxidation reactions were performed to improve conversion on a larger reaction scale. Fatty ester or jatropha oil was diluted using benzene (molar ratio of C=C on fatty ester/benzene 1/5) in a three-neck round bottom flask. H_2_O_2_ and formic acid (molar ratio C=C/H_2_O_2_/FA = 1:10:1) as oxidant were added dropwise to the flask in two steps (40 °C at 400 rpm). The first step was allowed to react for 12 h, followed by water layer removal. Subsequently, another batch of FA and H_2_O_2_ (molar ratio C=C/H_2_O_2_/FA = 1:10:1) was also added dropwise to the reaction mixture. After another 12 h, the water layer was removed followed by washing the solution using demineralized water via liquid–liquid extraction (5 times). Afterwards, benzene was removed using rotary evaporation. The resulted products were analyzed with ^1^H NMR and GC-MS.

### 2.3. Furan Functionalization Reaction

#### 2.3.1. Functionalization of Fatty Esters or Jatropha Oil

The epoxidized fatty ester (or epoxidized jatropha oil) and furfurylamine (molar ratio oxirane ring/furfurylamine 1/5) were mixed in a glass vessel. To assist the epoxidation reaction, LiBr (110 mol % for epoxidized fatty ester or 50 mol % for epoxidized jatropha oil, with respect to the mol of double bonds) were added to the mixture. Afterwards, the vessel was closed and sealed, to prevent evaporation of furfurylamine. The mixture was further heated at 80 °C, and mechanically stirred at 400 rpm for 24 h. The product was dried in vacuum oven at 50 °C to remove the excess of unreacted furfurylamine. The dried product was further dissolved in CHCl_3_, followed by liquid–liquid extraction using demineralized water to remove the LiBr salt. Afterwards, the chloroform was removed using rotary evaporation followed by drying in vacuum oven at 50 °C until constant weight achieved. The final products were analyzed with ^1^H NMR.

#### 2.3.2. Furan Functionalization of Alternating Polyketone

Polyketone-50 (50% ethylene content) was added to a sealed 250 mL glass reactor, equipped with an oil bath and a mechanical stirrer with a U-type propeller. The polyketone was preheated to the liquid state at 100 °C. Furfurylamine (equimolar amounts of 1,4-dicarbonyl groups and amine groups) was added, dropwise, in 10 min. The reaction proceeded for 4 h, with a stirring speed of 400 rpm. After the reaction, the mixture was washed several times with demineralized water to remove the unreacted furfurylamine. The product was filtered and freeze-dried, obtaining PK-Furan as a light brown powder. This procedure represents a slightly modified form of a published one [6,7].

### 2.4. Crosslinking Reaction

#### 2.4.1. Crosslinking Reaction of Furan-Functionalized Fatty Ester or Jatropha Oil

The furan-functionalized compound and bismaleimide were dissolved in a round bottom flask equipped with magnetic stirrer and a condenser. Afterwards, the reaction was carried out at 50 °C, 300 rpm, for 24 h. After chloroform removal, the product was dried in vacuum oven at 50 °C until constant weight achieved.

#### 2.4.2. Crosslinking of Furan-Functionalized Fatty Ester or Jatropha Oil with PK-Furan

PK-Furan and the furan-modified fatty ester or jatropha oil were reacted with an equimolar amount of bismaleimide (ratio of maleimide/furan groups—1:1) using CHCl_3_ (10 wt %) as solvent, in a round bottom flask equipped with magnetic stirrer and condenser. The fatty acid derivative contained 10% or 20% of the total furan groups. The reaction was carried out at 50 °C and 300 rpm for 24 h. The product was dried in a vacuum oven at 50 °C until CHCl_3_ was removed.

### 2.5. Characterization

#### 2.5.1. ^1^H NMR

Characterization of the products by ^1^H NMR was performed by using a Varian Mercury Plus 400 MHz (Mundelein, IL, USA) using CDCl_3_ or DMSO-d_6_ as solvents. MestRenova software (Santiago de Compostela, Spain) was used for NMR spectra processing. The different peaks were assigned according to previously published results [10]. The reaction conversion of epoxidation of methyl oleate was calculated from the ratio between peak area of epoxide peak formed in epoxidized methyl oleate (–CHOCH– proton 2.8 ppm) towards the initial peak of the double bond from methyl oleate (–CH=CH– proton 5.4 ppm), which is defined as

(1) $$Conversion\left(\%\right)=\frac{A_{epoxide}}{A_{C=C}}\times 100$$

The calculation for epoxidation conversion of methyl linoleate also follows the formula presented in Equation (1). However, the peak used for the calculations represents 4 protons (two double bonds) each, instead of 2 for methyl oleate. The peak area of formed epoxides in epoxidized methyl linoleate (–CH < proton 3.1 and 3.2 ppm) were compared towards the initial peak of the double bond from methyl linoleate (–CH < proton 5.4 ppm).

Jatropha oil consists mainly of oleic and linoleic chains, attached to a glycerol. Peaks belonging to the glyceride are present at 4.2 and 4.3 ppm (–CH_2_–), representing 4 protons, and 5.3 ppm (–CH<), represent 1 proton which overlaps with the double bond signal. The signals of the overlapped double bond on the triglyceride were calculated by difference, based on the integration of other non-overlapped peaks (–CH_2_–). The epoxide signals appear at 2.8 to 3.2 ppm, similar to the oleic and linoleic products. Therefore, the conversion of the reaction is also calculated following Equation (1).

#### 2.5.2. GC-MS

Epoxidized fatty acid was analyzed using THF as solvent. Fatty acid composition of jatropha oil was analyzed as its transmethylated derivates [22]. For sample preparation, 100 mg of jatropha oil was dissolved in *tert*-butyl ether (5 mL). A sample of 130 μL was mixed with 70 μL of trimethylsulfonium hydroxide inside a 2 mL autosampler vial. The vial was capped and properly shaken to ensure mixing before it was measured using a gas chromatography-mass spectrometry (GC-MS) in a Hewlett Packard (HP, Palo Alto, CA, USA) 5890 series II Plus with HP Chemstation G1701BA, B0100/NIST library software and a Hewlett Packard (HP) 5972 series mass selective detector. Fused-silica column (Solgel-1MS column: 30 m × 0.25 mm × 0.25 μm) was used as the gas carrier at a column pressure of 49 kPa. The column temperature was 80 °C (hold 4 min), 80–150 °C (rate 10 °C/min), 150–240 °C (rate 3 °C/min), 240 °C (hold 10 min). After that, the temperature was further increased at 3 °C/min to 240 °C, and held for 10 min. A split ratio of 1:100 was used, the injector and detector temperature were set at 280 °C whereas the gas flow rate was set to 1 mL/min.

#### 2.5.3. DSC

Analysis of thermoreversible crosslinking was performed using differential scanning calorimetry (DSC). Samples of 10 to 15 mg were placed in an aluminum pan. After sealing the pan, the temperature was raised at 5 °C/min, from a starting temperature to a maximum temperature. The samples were kept at the maximum temperature for 1 min. After cooling down, the cycle was repeated for an overall of 4 cycles. The output was given as the heat flow in the system against temperature. All first cycles of the DSC were removed from the thermogram, since it mostly represents thermal history of the sample. Therefore, second cycles were further referred to as first cycles, and so on.

#### 2.5.4. DMTA

Dynamic mechanical thermal analysis was achieved using a Rheometrics Scientific solid analyzer (RSA II, Midland, ON, Canada). Analysis was conducted in the dual cantilever mode with a strain rate of 0.025% and an oscillation frequency of 1 Hz. The distance between the outer set points was 35 mm. The sample was heated from room temperature to 120 °C, with a heating rate of 3 °C/min. Samples needed for the DMTA measurements were powdered in a coffee grinder, and pressed in rectangular bars at 120 °C and 4 MPa for 20 min, with a length of 54 mm, a width of 6 mm, and a thickness of 1.5 mm.

**Figure 1:**
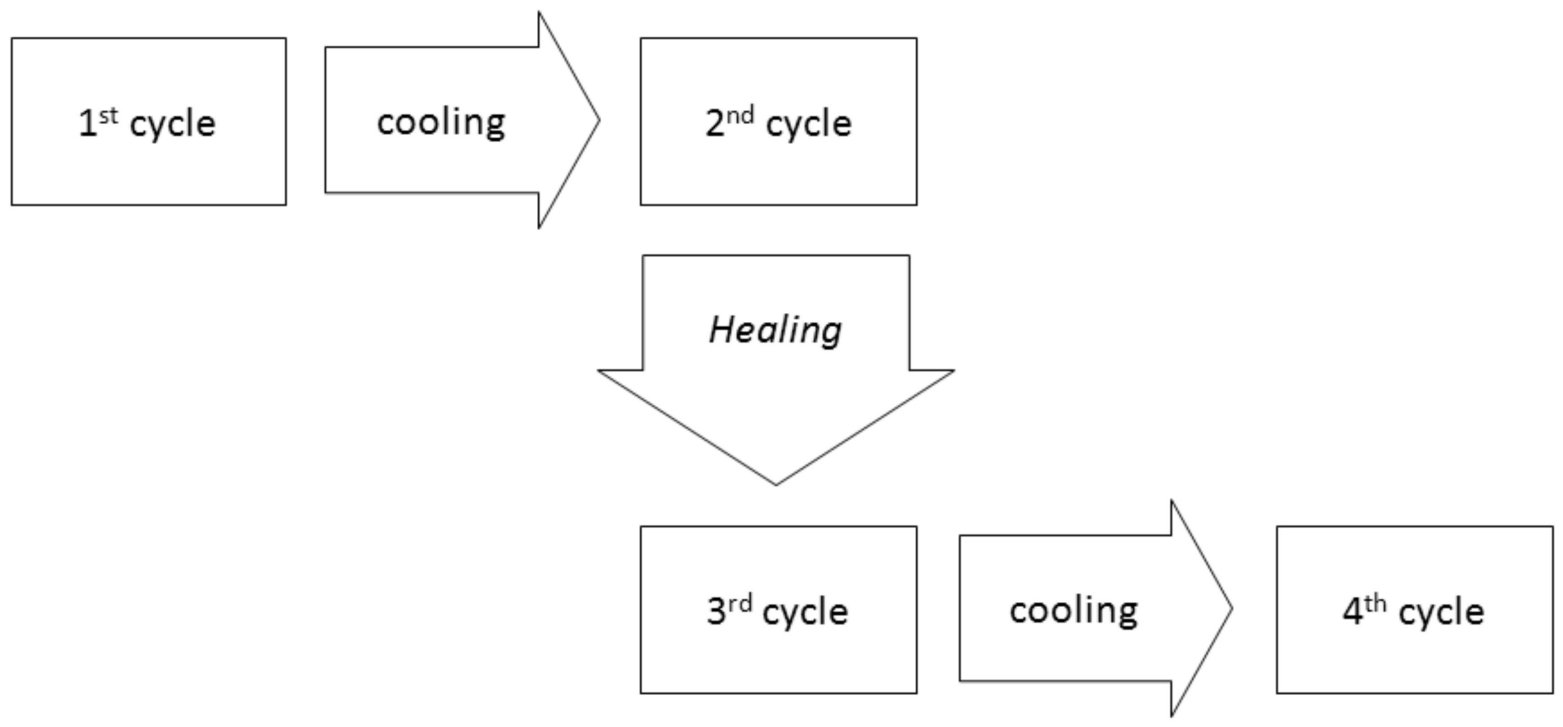
Sequence of DMTA analysis.

For the analysis, the bars were clipped between the screws. The outer screws were kept in place with a distance of 35 mm, and the inner screw was moved up and down with a strain rate of 0.025% and a frequency of 1 Hz in the dual cantilever mode. The temperature was raised from room temperature to 120 °C, with a heating rate of 3 °C/min. The output exists out of a loss modulus, storage modulus, and the tan(δ) against temperature. The loss modulus or elastic modulus (*E*’) shows the ability of the product to return to its initial state. It is also referred to as the material’s “stiffness.” The storage modulus (*E*’’) represents internal strain and viscous forces, a measure of deformation. The ratio of the loss modulus to the storage modulus gives the dampening property, tan(δ). It relates to all of the molecular movement and phase transitions. For example, a peak of tan(δ) is found at the *T*_g_ for a polymer. The DMTA analysis was divided into two steps. Two cycles were done consequently per sample, only allowing the system to cool between the two runs. Afterwards, the sample was “healed” for 24 h at 50 °C, to ensure bond reconstitution, followed by another two cycles of measurements (Figure 1).

## 3. Results

### 3.1. Furan-Functionalized Methyl Oleate and Methyl Linoleate-Based Polymers

**Scheme 1:**
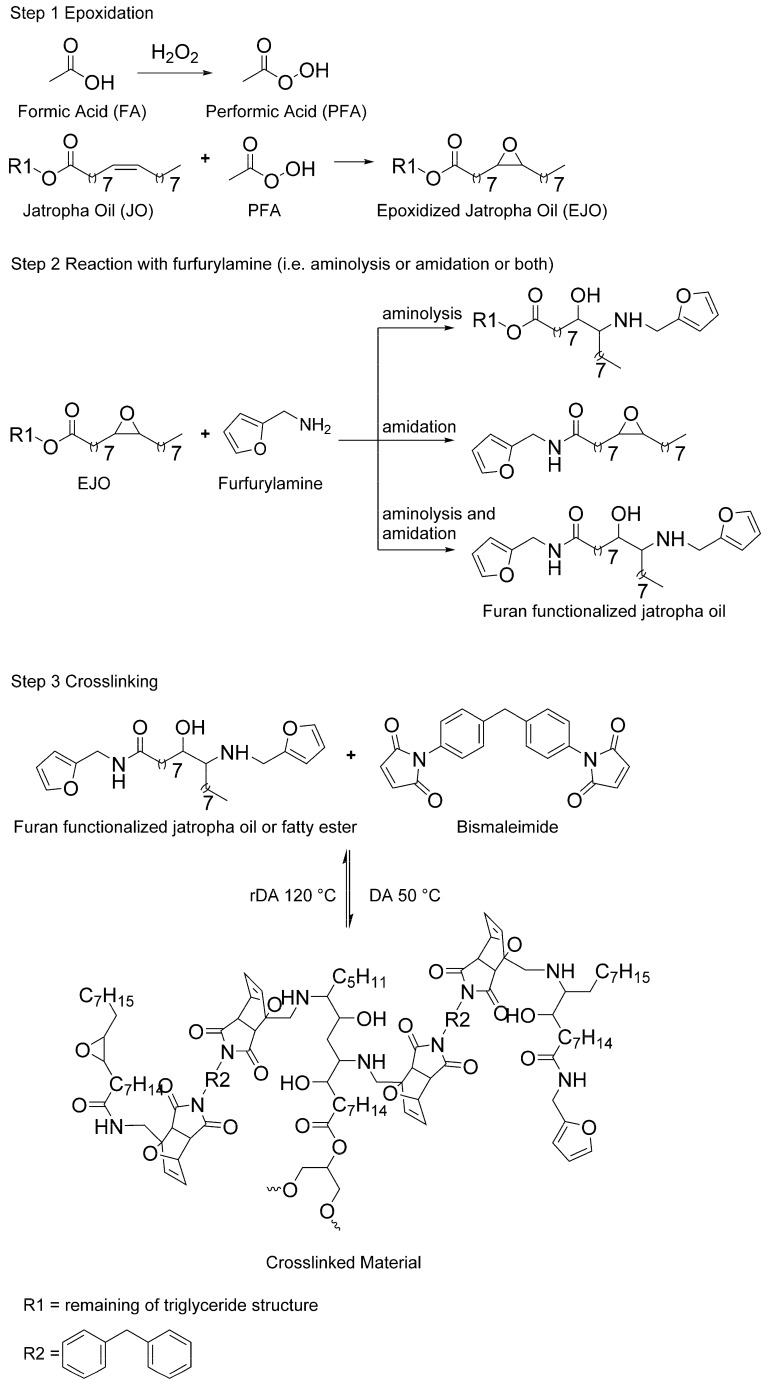
Reaction scheme of jatropha oil-based Diels–Alder-crosslinked polymer.

This section discusses the use of methyl oleate (MO) and methyl linoleate (LO) as a preliminary study, with respect to further study of jatropha oil (JO) as a crosslinked polymer precursor. Even though the fatty acid content of JO may vary depending on species, climate, soil quality, etc., MO and LO are chosen based on the fact that both components are the major fatty acid constituents in JO [9,10]. As the first step of the polymer synthesis, MO and LO were epoxidized according to published procedures [15]. Formic acid was reacted in situ with H_2_O_2_ to form performic acid, which acts as an oxygen donor for the alkene. The donated oxygen is replenished by H_2_O_2_, generating water (Scheme 1). Formic acid can also open the oxirane ring, and hydrolysis can occur in the presence of water. Benzene has been added as diluent of the organic phase (i.e., fatty esters) to minimize oxirane ring opening [23]. Since the two layers are immiscible, the degree of mixing has a large influence on the reaction. However, at a certain point, a high degree of mixing may promote the unwanted oxirane ring degradation process. In addition, higher reaction temperatures generally favor the degradation reaction [15,16].

The opening of the oxirane ring may occur in the presence of water and formic acid and, to a lesser extent, H_2_O_2_. High chemical activity of formic acid may cause depletion of oxygen in the system if the H_2_O_2_ is no longer present, resulting in lower conversions [15,16]. Therefore, two-step epoxidation reactions were formulated to ensure the presence of H_2_O_2_. As expected, the epoxidation reaction of MO and LO gives full conversion (NMR spectra not shown for brevity) and, also, good yield (i.e., MO: 76% and LO: 92%). In addition, full conversion gives one oxirane group for epoxidized methyl oleate (ep-MO), and two groups for epoxidized methyl linoleate (ep-LO). This number is rather important for further steps, since the oxirane ring acts as the basis for the furan functionalization reaction.

**Figure 2:**
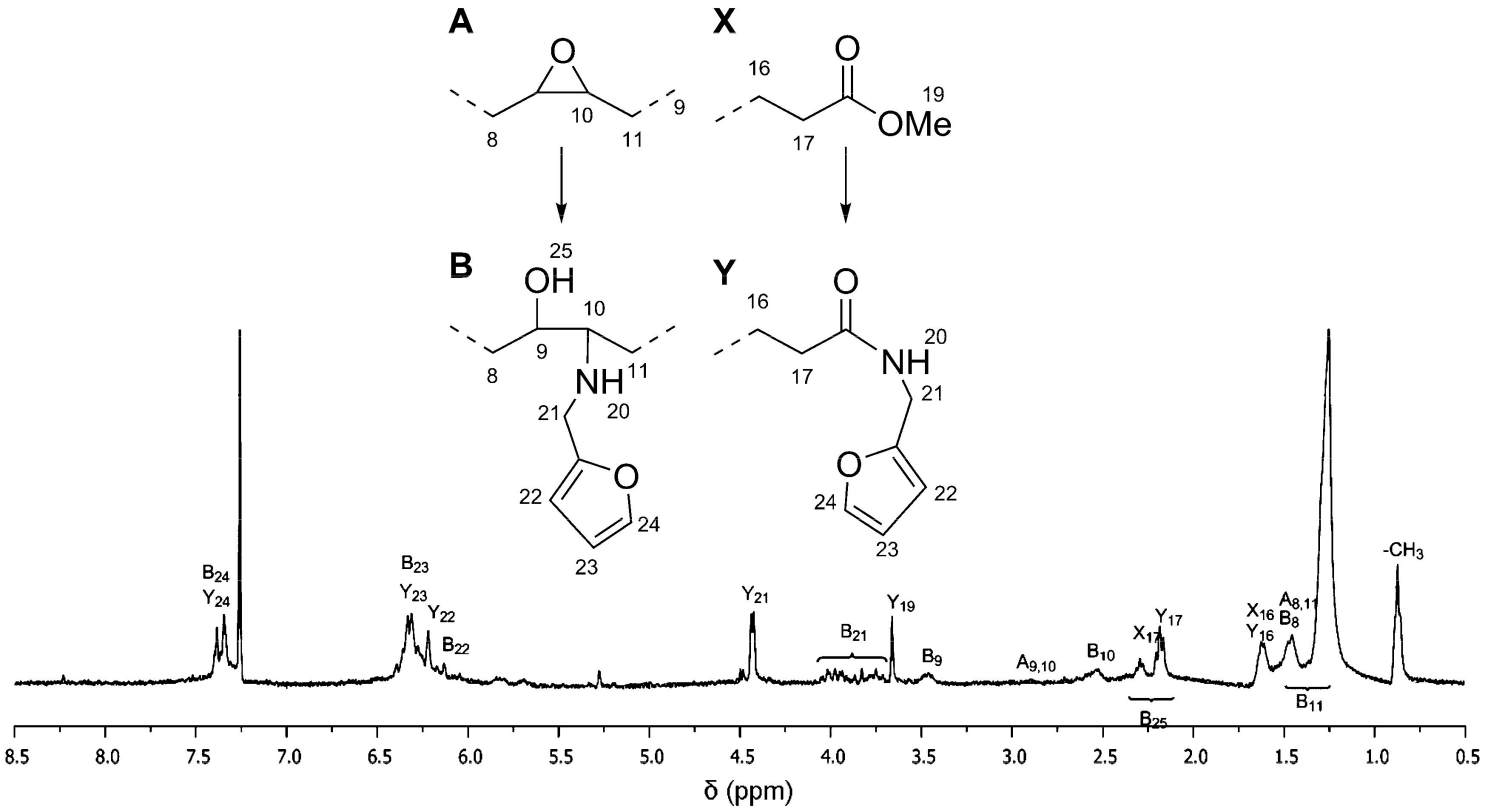
^1^H NMR-spectrum of FAO with functional groups assigned.

To the best of our knowledge, the reaction between furfurylamine and epoxidized fatty ester has not been reported in literature. The reaction does not spontaneously take place at atmospheric pressure and room temperature. Since the primary amine of furfurylamine molecule is rather weak to undergo ring opening reaction with the oxirane group, this aminolysis reaction requires assistance in the form of a catalyst. In addition, the catalyst is also expected to assist amidation reaction on the ester groups of the epoxidized fatty esters (Figure 2). Both reactions are rather important, especially for ep-MO, since it requires at least two furan groups in order to assemble a polymeric chain.

Based on a similar aminolysis procedure with different primary amines described in literature [15,18], oxirane contained ep-MO or ep-LO was reacted with furfurylamine. After 24 h, the reaction gives a conversion of 82% on the ester group and 89% on the epoxide for ep-MO. While ep-LO has a conversion of 83% on the ester group and 75% on the epoxide (confirmed by NMR). The presence of two oxirane ring close to each other on ep-LO is believed to be responsible for lower aminolysis conversion (steric hindrance). These partial conversions are actually desired for later reactions with JO (see below). In addition, the functionalized oleic (FAO) and furan-functionalized linoleic (FALO) chains have approximately 1.7 and 2.4 furan groups/molecule respectively. The mixtures of partially converted FAO (including also stereo-isomers) display a broad range of proton signals in ^1^H NMR spectra. Therefore, signal assignment is highlighted only to the important peaks (Figure 2).

**Figure 3:**
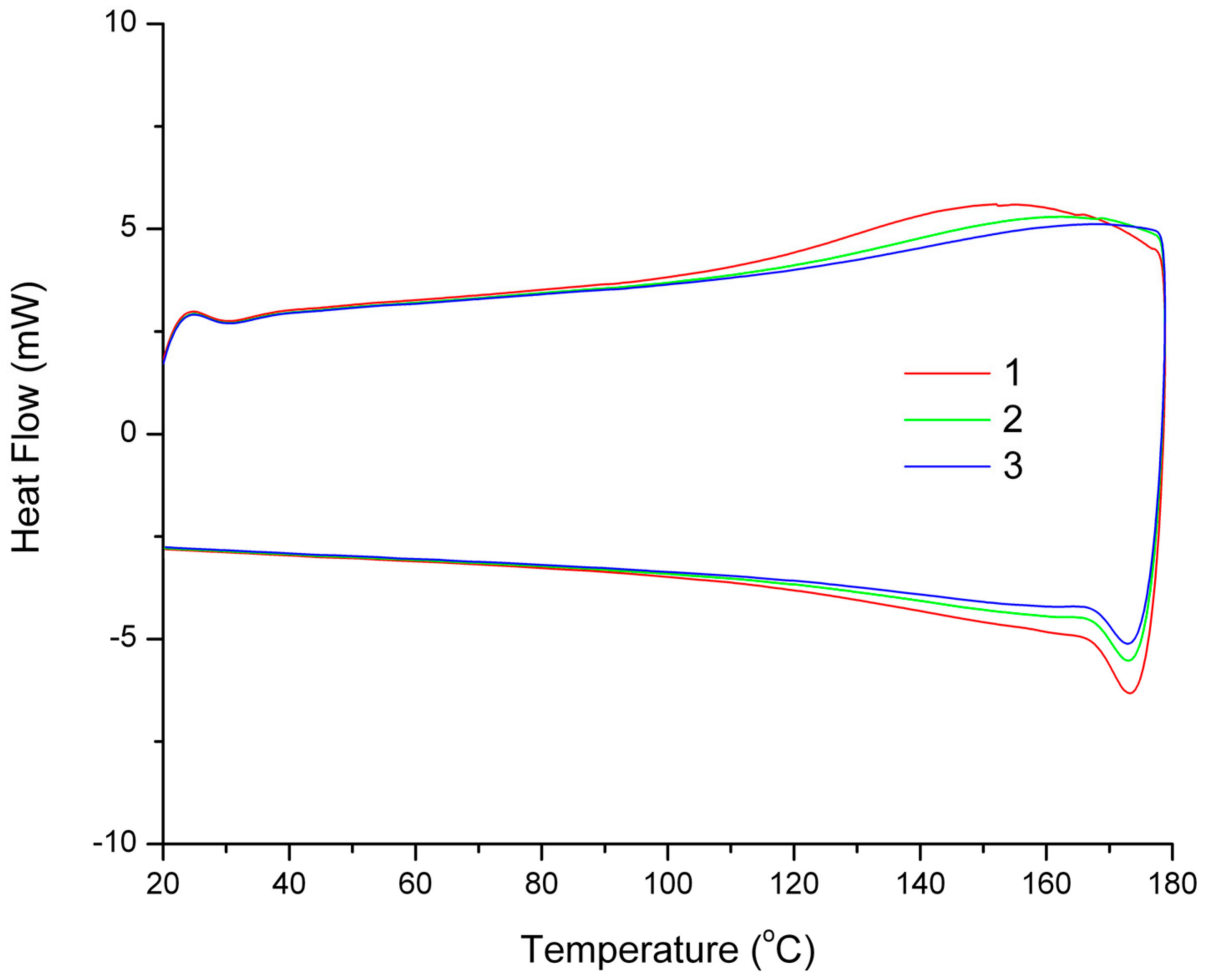
DSC thermogram of cr-FAO. (25 °C → 180 °C for 1 min, 10 °C/min, 3 cycles).

As preliminary study, FAO was reacted with bismaleimide to obtain polymer as a result of the DA adduct formation. With a maximum of two possible adduct site per FAO molecule, the reaction may only give a linear chain. However, poor solubility of the resulted polymer cr-FAO in some solvents (i.e., acetone, THF, chloroform, and DMSO) at room temperature indicates a relatively high molecular weight product was obtained. The DA-rDA thermoreversibility system on cr-FAO was investigated using DSC (Figure 3). At 110 °C, bonds rupture via rDA reaction route starts taking place indicated by heat uptake. Each consecutive cycle reveals the decrease on the material “thermo-recovering degree” specified by the loss of energy uptake.

The DA-rDA system is an equilibrium system with respect to temperature and also a time-dependent mechanism, especially at lower temperature region. The decrease on thermo-recovering degree most likely caused by the partial failure on molecular reconstitution via DA reaction at given time. The presence of more stable DA adduct isomer (i.e., *endo* and *exo* adduct) also contribute to the drop of thermo-recovering degree, as previously reported for similar systems [7]. In addition, loss of functionality through permanent crosslinking also arises as another possibility. The latter possibility only implies if reduction of weight occurs. The possibility can be eliminated since the thermogram profile at 40 °C to 80 °C shows no apparent decrease of heat uptake. The crosslinking reaction of FALO and bismaleimide (*I*_m/f_ = 1) gives much brittle product compared to cr-FAO. The difference caused by the number of possible furan functionality to undergo DA reaction with bisamleimide. A crosslinked product was obtained since the average furan groups per molecules are more than two.

### 3.2. Furan-Functionalized Jatropha Oil-Based Polymers

**Figure 4:**
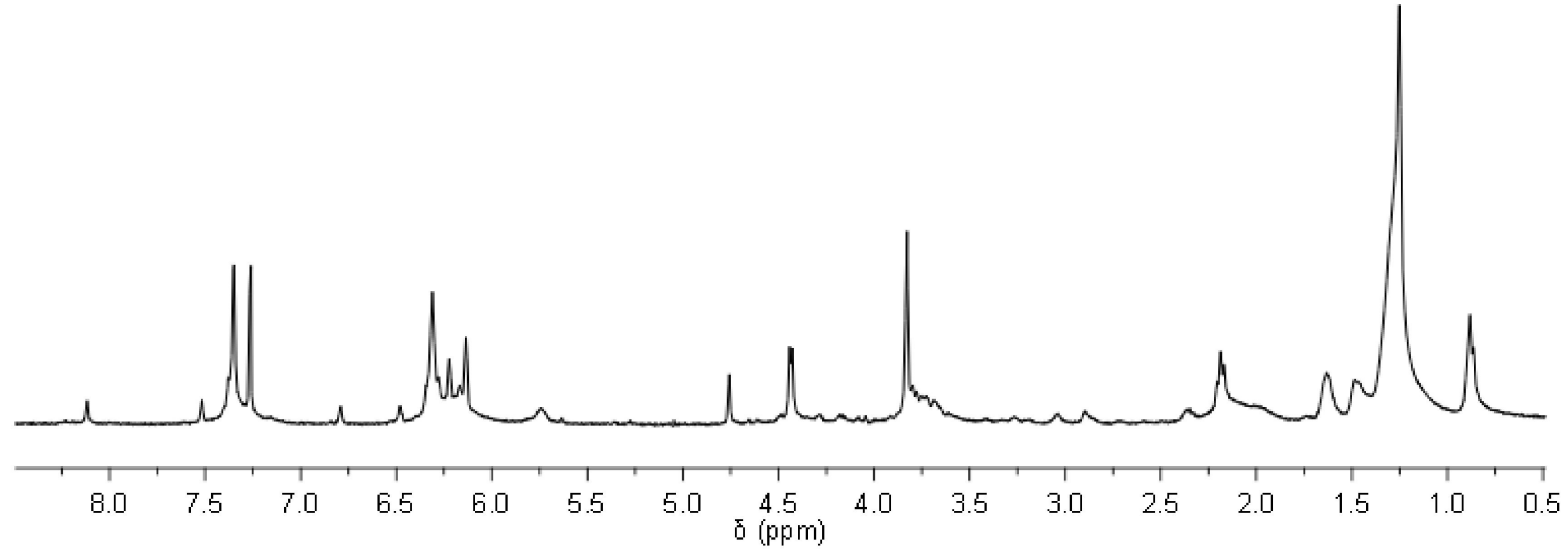
^1^H NMR spectra of furan-functionalized jatropha oil (400 MHz; CDCl_3_).

Through same method used on epoxidation of the fatty esters (i.e., MO and LO), JO was fully epoxidized resulting high yield of 96% (Figure 4). Therefore, the full conversion gives an average of 3.3 epoxides group per triglyceride molecules of ep-JO. As mentioned earlier, this sum of oxirane ring per molecules is important since it will undergo aminolysis reaction with furfurylamine as nucleophile. In addition, occurring amidation reaction on the ester groups splits the fatty ester from the original triglyceride molecule.

Reactions of ep-JO with furfurylamine were performed with lower LiBr intake (i.e., 50%). Partial amidation on furan functionalization reaction of ep-JO result in a broad variety of Furfuryl-amine functionalized Jatropha Oil (FAJO) products (i.e., furan-functionalized mono-, di-, triglyceride, and free fatty amide). The multiplet at 3.7 ppm gives strong indication of glycerol or monoglyceride (Figure 4). However, some signal overlap occurs since aminolysis products give a broad signal between 3.7 to 4.0 ppm. Steric hindrance of the triglyceride structure contributes to partial aminolysis reaction, with remaining unreacted epoxide peaks at 2.9 to 3.2 ppm. A triglyceride contains three ester groups close together making the reaction site bulky for amidation. The stereochemistry of the molecule hinders its accessibility to nucleophile (i.e., furfurylamine) as well as LiBr to assist the reaction. Improving reaction conversion by using an excess of LiBr at certain point might lead to contra-productive results since the metal ion may also interact with two carbonyl groups through a chelation effect [20]. The amide signal at 4.4 ppm is used to calculate the amidation reaction conversion, while the remaining oxirane group is used for the aminolysis. A total of 4.2 furan groups were found for every triglyceride as combined reaction of amidation (47% conversion) and aminolysis (74% conversion).

**Figure 5:**
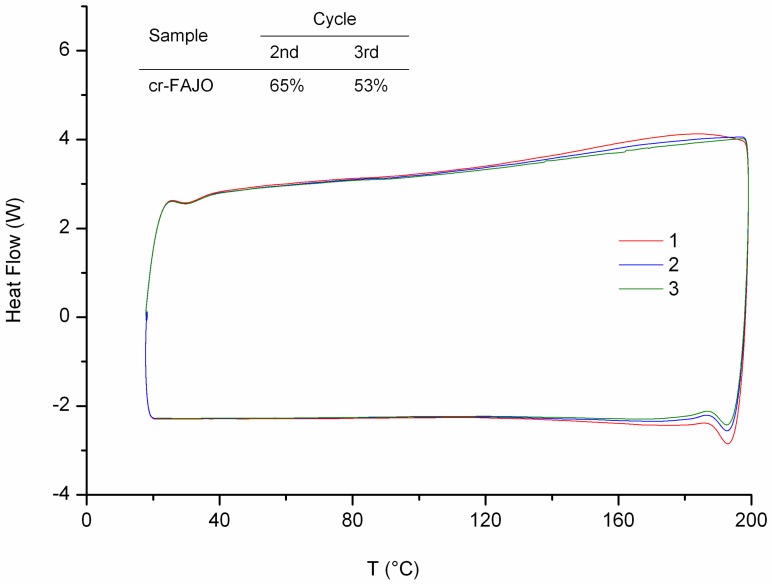
DSC cr-FAJO with its thermoreversibility profile, *I*_m/f_ = 1 (20 °C → 200 °C for 1 min, 10 °C/min, 3 cycles).

Crosslinking reactions of FAJO using bismaleimide (equimolar ratio) were performed in a reflux setup. The resulted product (cr-FAJO, *I*_m/f_ = 1) was analyzed using DSC to investigate its thermoreversibility profile (Figure 5). An increase of heat flow starts to occur around 125 °C for each cycle which represents the rDA reaction. Similar to cr-FAO and cr-FALO, the thermo-recovering degree of each cycles decrease gradually compared to its previous cycles. The decrease can be explained using same possibilities as for crosslinked fatty esters (i.e., short time for bond reconstitution and formation of most thermodynamically stable adducts isomer) [6,7]. In addition, relatively high degree of crosslink on cr-FAJO caused the product to have a brittle nature.

### 3.3. Crosslinking of Furan-Functionalized Fatty Esters or Jatropha Oil with PK-Furan

**Figure 6:**
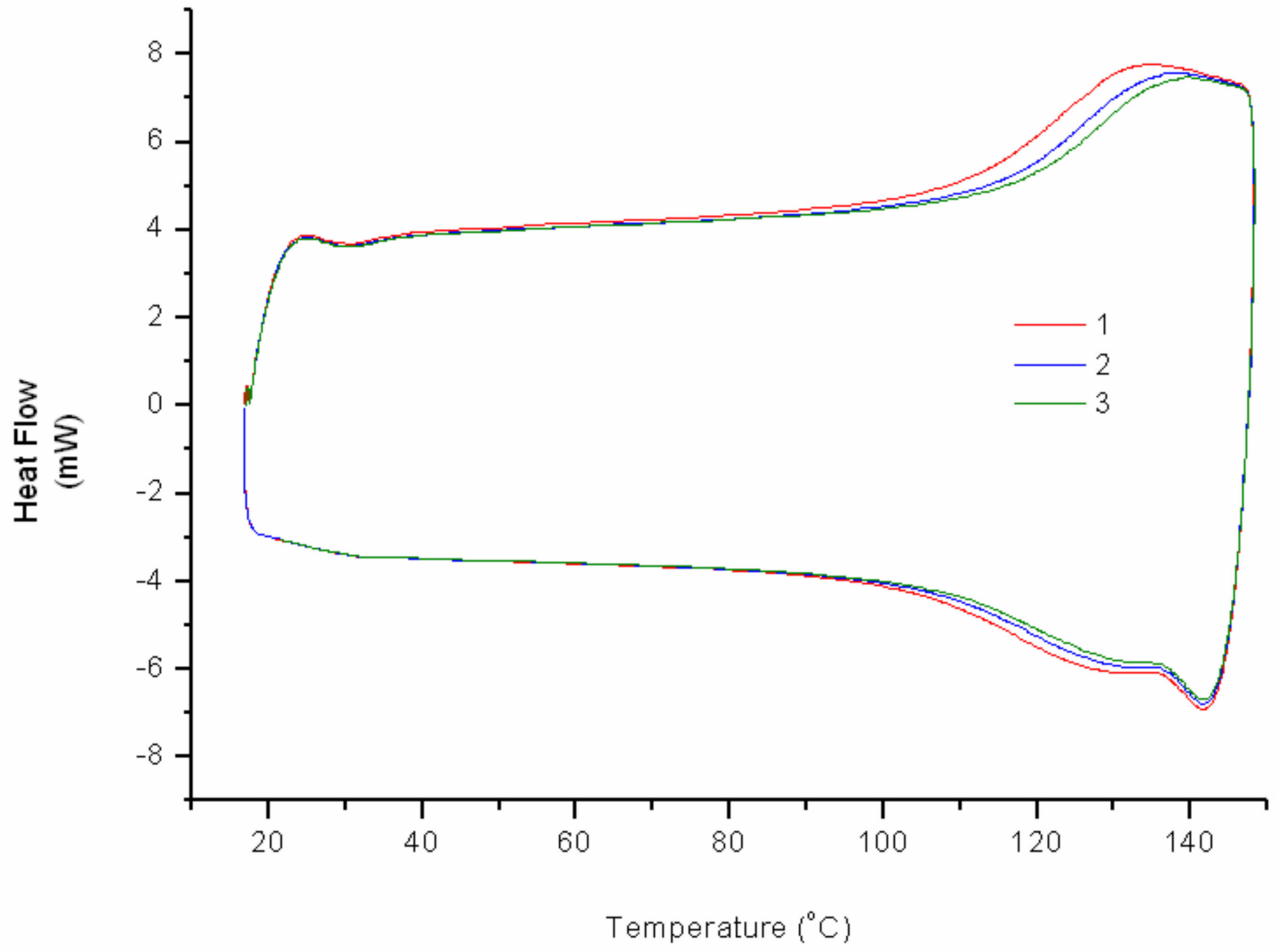
DSC of FAJO-10% in PK-Furan (20 °C → 150 °C for 1 min, 10 °C/min, 4 cycles).

The use of furan-functionalized fatty ester or jatropha oil, for partial substitution in well-established PK-Furan thermoreversible polymers, is intended to give improvements to polymer properties and, also, an added value through use of renewable resources. Thermoreversibility of PK-Furan, as reference material, and several poly blends with FAO, FALO, and FAJO, are investigated using DSC (Table 1). Presented values are the results of normalization with respect to its previous cycle. In addition, for the sake of brevity, only DSC profiles of FAJO-10% in PK-Furan are presented (Figure 6).

A constant decrease in thermoreversibility can be observed on every sample. The thermos-recovery degree of mixture samples is higher with respect to pure PK-Furan, except for the sample with 10% furan-functionalized linoleate (cr-FALO-10%). This effect can be attributed to the DA adduct bond density of each sample. The samples were mixed based on the percentage of total furan groups reacted. Therefore, the amounts of furan groups per unit weight or volume may differ. For FALO and PK-Furan, the numbers of furan groups per gram are similar (i.e., 4.62 × 10^−3^ and 4.58 × 10^−3^ mol/g, while the furan groups content for FAO and FAJO are, respectively, 79% and 68%, compared to PK-Furan. Therefore, the system with higher adduct density will require more energy to decrosslink and, indeed, more time to fully reconstitute. However, it should be highlighted that the DA adducts in PK-Furan samples and furan-functionalized fatty esters have a different fundamental structure. The DA adducts of furan-functionalized fatty esters are similar to crosslinking points in epoxy resin. On the other hand, DA adducts of PK-Furan system eventually form a crosslinked structure of relatively long backbone on alternating polyketone. Therefore, the adduct reconstitution process in PK-Furan will be easier and faster, since the rDA route will leave the polyketone backbone intact. The addition of furan-functionalized compounds will eventually increase the free space on PK-Furan polymer networks. Therefore, heat would penetrate more easily and improve the thermo-recovering efficiency, especially demonstrated by increasing the FAJO content to 20%. On the other hand, the addition of FALO will create a very dense DA adduct network. These dense networks may hinder the reconstitution process from taking place (steric hindrance). Therefore, the cr-FALO-10% sample shows lower efficiency with respect to pure cr-PK-Furan.

The mechanical properties of the samples were evaluated using DMTA, following the works of Zhang with PK-Furan [6]. It is found that the second cycle of DMTA measurement of PK-Furan slightly differs from the first one. However, subsequent cycles show no significant differences in the mechanical properties. In addition, after the healing process, the mechanical properties were found to be similar to the first measurement. Therefore, it is possible that the difference may be caused by partial “healing”. To overcome this, we used in our investigation a two-steps measurement strategy. Each step consisted of two cycles of DMTA measurement, and a “healing” process was applied before the second step was conducted.

**Figure 7:**
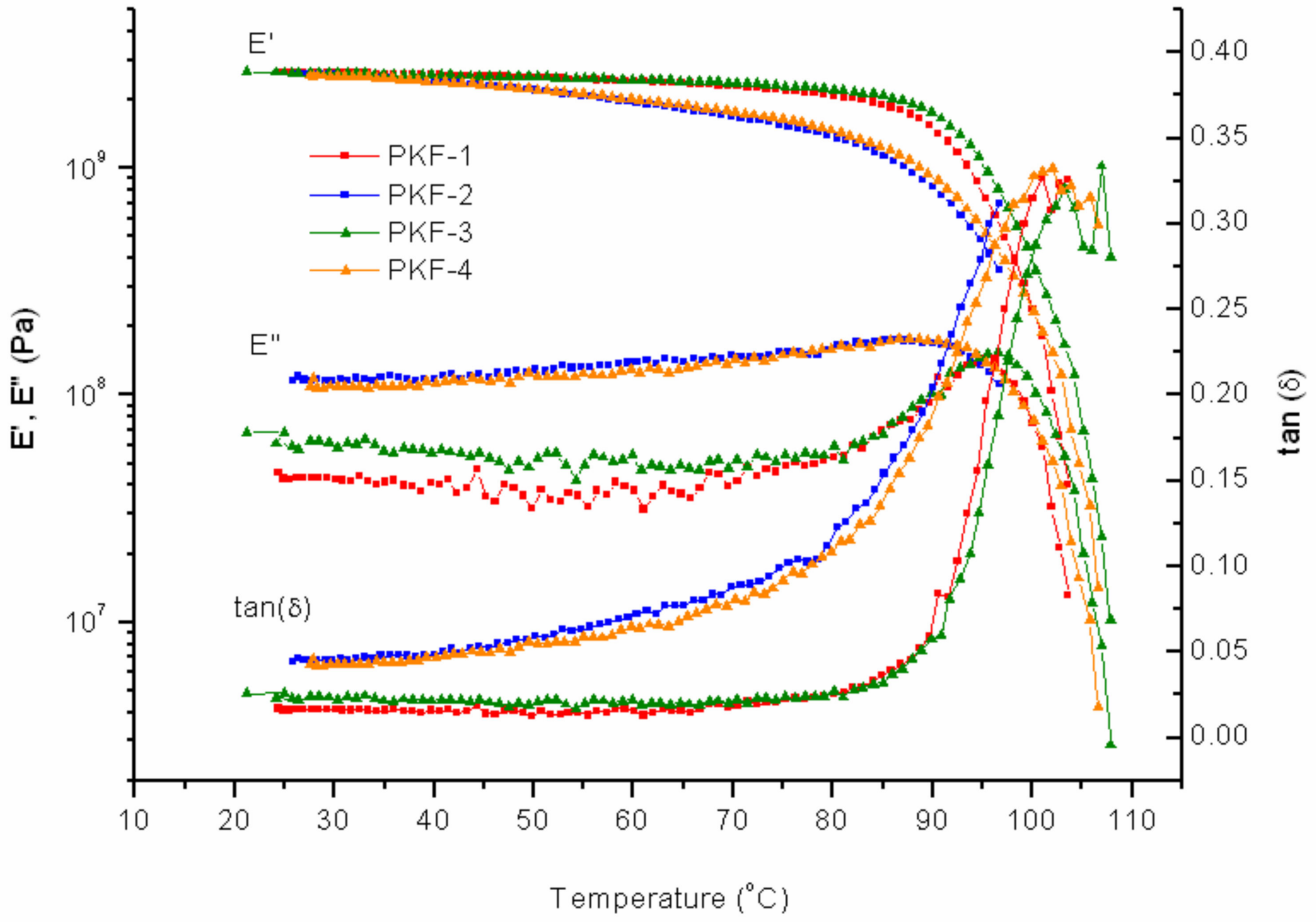
DMTA-graph of PK-Furan; 2 cycles before healing (PKF-1 and PKF-2) and after healing (PKF-3 and PKF-4).

On the first cycle of PK-Furan, the increase in temperature does not affect the elastic storage modulus (*E*’’) in a significant way, up to 70 °C (Figure 7). Due to the high degree of crosslinking (*I*_m/f_ = 1), the viscosity effects, which are normally caused by the increase of temperature, are greatly reduced. A temperature increase up to 95 °C, shows an exponential increase of *E*’’. Further temperature increase eventually increases the viscous forces, followed by rDA bond ruptures, which reduce the crosslinking and cause deformation. Eventually, the DMTA measures no resistance from the sample with the given strain rate. After the first cycle, the system was cooled down and the measurement was repeated. The initial storage modulus is increased, and its slope is constant. Decrosslinking and deformation effects that occurred in the first cycle become the initial state of the second cycle. The mechanical properties are more dependent on the temperature due to less crosslinking. The same conclusions can be derived from the difference in the curves of tan(δ). The same measurements were repeated after the healing process. Cycles 3 and 4, after the healing process, show similar results compared to cycles 1 and 2, showing a fully healed product (Figure 7). The results are in agreement with previously reported literature [5,6].

**Figure 8:**
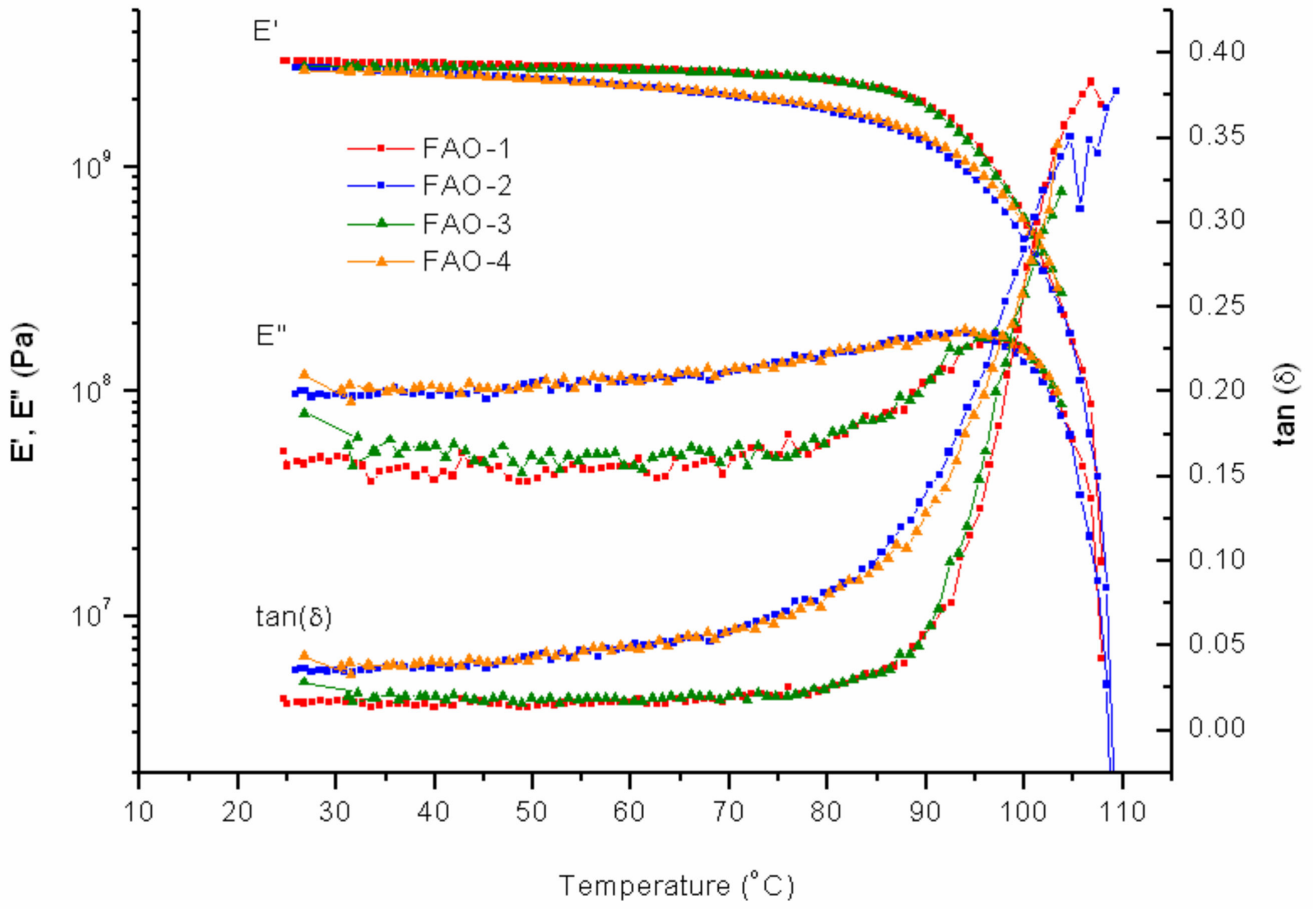
DMTA-graph of FAO-10% in PK-Furan; 2 cycles before healing (FAO-1 and FAO-2) and after healing (FAO-3 and FAO-4).

The addition of FAO-10% to PK-Furan does not result in significant changes to the mechanical properties, compared to pure PK-Furan. Measurements after 24 h of healing process also showed full self-healing properties (Figure 8).

**Figure 9:**
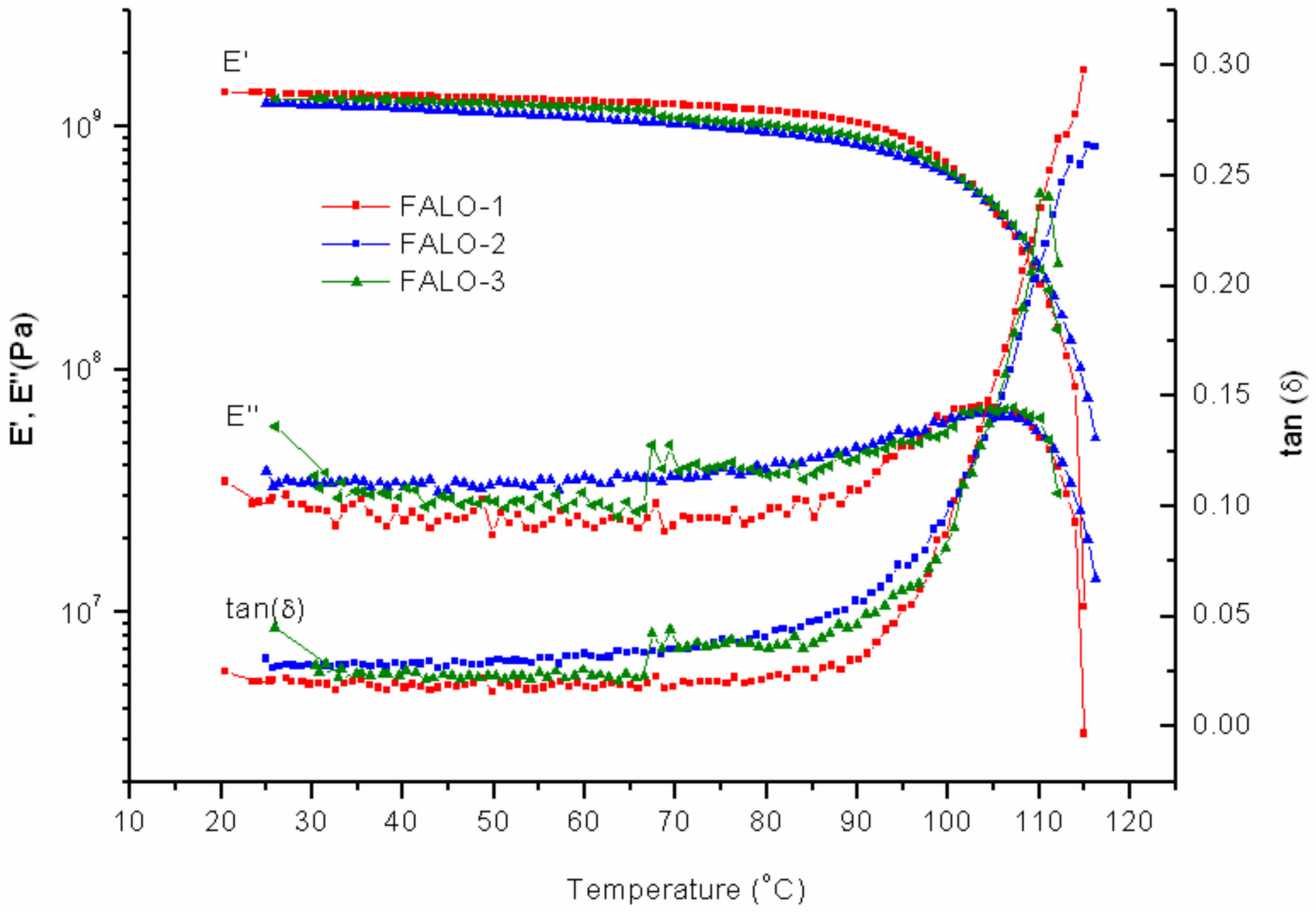
DMTA-graph of FALO-10% in PK-Furan; 2 cycles before healing (FALO-1 and FALO-2) and after healing (FALO-3).

As expected, samples with FALO-10% intake were more fragile compared to any others. The multifunctional furan-functionalized linoleic, resulting in very dense DA adduct, contributes to the brittleness of the networks. From the DMTA measurement, the modulus shows lower values with respect to those of FAO and PK-Furan (Figure 9). There seems to be a smaller difference between the first and the second cycle. The reduced peak of the storage modulus in the second cycle implies a difference in molecular state. A minor increase of the *E*’’ in the second cycle up to 85 °C implies less deformation within the network. Interestingly, a difference with PK-Furan is found when looking at the effect of temperature on the material. The peak of the storage modulus lies higher in temperature than that of PK-Furan, and the decrease in strength for both the loss and the storage modulus shows a less steep slope for FALO, compared to PK-Furan. The result shows that FALO-10% exceeds the PK-Furan in decrosslinking temperature. The third cycles gave an increase to the storage modulus at 67 °C, even if the effect seems small on the loss modulus. The storage modulus peak was improved compared to cycle 2, showing some healing of the product. However, the sample could not regain its initial mechanical properties. Consequently, none of the samples could survive another cycle of measurement.

**Figure 10:**
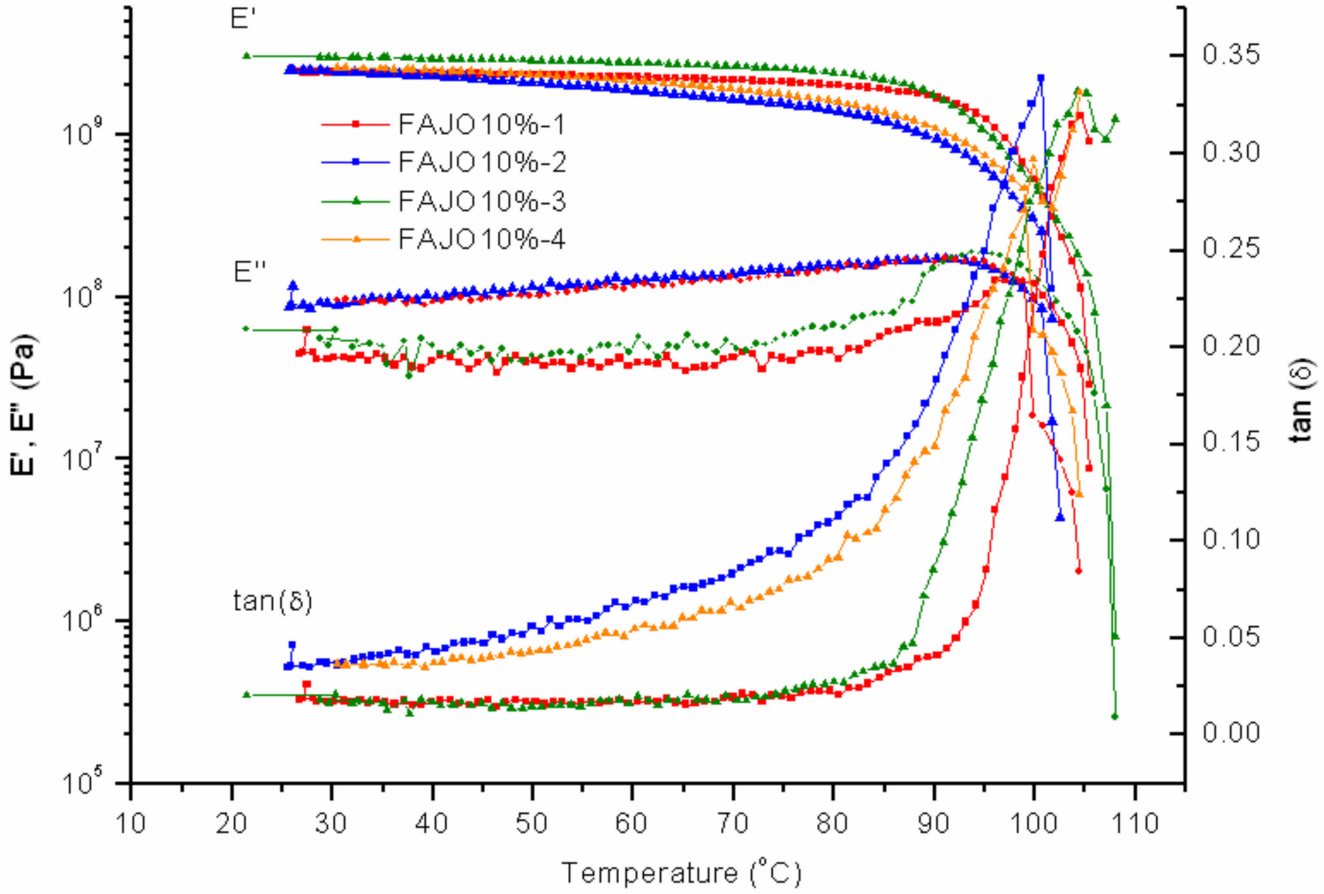
DMTA-graph of FAJO-10% in PK-Furan; 2 cycles before healing (FAJO-10%-1 and FAJO-10%-2) and after healing (FAJO-10%-3 and FAJO-10%-4).

FAJO was mixed with PK-Furan in two different fractions, 10% and 20%. Compared to PK-Furan, the results of FAJO-10% yield no large differences before healing (Figure 10). A difference can be observed in the storage modulus between the first cycle and third cycle. However, the differences were not further observed after the fourth cycles.

**Figure 11:**
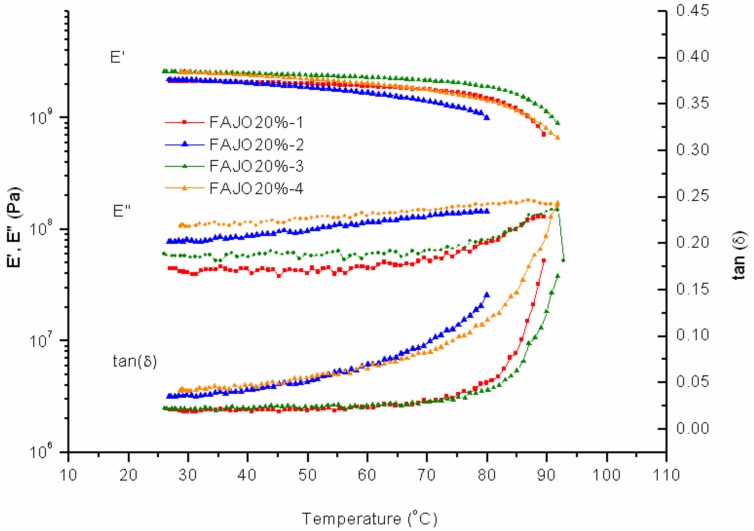
DMTA-graph of FAJO-20% in PK-Furan after healing; 2 cycles before healing (FAJO-20%-1 and FAJO-20%-2) and after healing (FAJO-20%-3 and FAJO-20%-4) be divided by subheadings. This should provide a concise and precise description of the experimental results, their interpretation, as well as the experimental conclusions that can be drawn.

Increasing the amount of FAJO up to 20% gives a more resulting fragile material. At low temperature measurement of the first two cycles, there is no significant resistance on the sample (Figure 11). The first cycle shows a peak for the storage modulus at 90 °C, whereas PK-Furan showed that peak at 95 °C. The second cycle shows the sample’s initial state has changed. The third cycle shows similarity with the initial mechanical properties (first cycle). The fourth cycle shows a similar profile to the second cycle with higher measured temperature (i.e., up to 90 °C). The intake of 10% FAJO in PK-Furan does not significantly change the mechanical properties with respect to pure PK-Furan. This result is already considered as an improvement in terms of the (partial) use of renewable resources. However, higher FAJO intake (i.e., 20%) does not lead to better mechanical properties (i.e., brittle material).

## 4. Conclusions

Fatty acids capable of thermoreversible crosslinking were obtained by a three-step synthesis. Methyl oleate, methyl linoleate, and jatropha oil were epoxidized, furan-functionalized with furfurylamine, and crosslinked with bismaleimide, resulting in thermoreversible networks. Epoxidation of the fatty esters gave full conversion using a split method for the addition of reactants on larger scales. Solvent-free reaction of the epoxidized compounds with furfurylamine gave partial conversion of both ester and epoxide group. The furan-functionalized compounds were reacted with bismaleimide through a Diels–Alder reaction mechanism, forming a solid material. The furan-functionalized fatty esters were mixed with PK-Furan, aiming at the use of renewable sources components in a thermally reversible network. All samples showed full self-healing properties. Similar rDA temperature and faster thermoreversible kinetics were found for similar crosslinking densities. However, multiple unsaturated chains (i.e., linoleate) highly contribute to the brittleness and the failure of network reconstitution. Introduction of 10% of furan-functionalized linoleate in PK-Furan exceeds the reference PK-Furan in temperature stability thermoset and decrosslinking temperature.

## Figures and Tables

**Table 1:** Summary of thermoreversibility properties for furan maleimide-crosslinked samples (*I*_m/f_ = 1).

Sample	%_furan fatty ester/JO_	Cycle 2	Cycle 3
cr-PK-Furan	-	83%	83%
cr-FAO-10%	10	88%	89%
cr-FALO-10%	10	75%	82%
cr-FAJO-10%	10	84%	91%
cr-FAJO-20%	20	92%	93%
